# Spatial and Temporal Patterns in the Seasonal Distribution of Toxic Cyanobacteria in Western Lake Erie from 2002–2014

**DOI:** 10.3390/toxins7051649

**Published:** 2015-05-12

**Authors:** Timothy T. Wynne, Richard P. Stumpf

**Affiliations:** National Centers for Coastal Ocean Science, National Oceanic and Atmospheric Administration, 1305 East-West Highway, Silver Spring, MD 20910, USA; E-Mail: richard.stumpf@noaa.gov

**Keywords:** harmful algae, cyanobacteria, ecological forecasting, Lake Erie, remote sensing, MODIS, MERIS, blue-green algae

## Abstract

Lake Erie, the world’s tenth largest freshwater lake by area, has had recurring blooms of toxic cyanobacteria for the past two decades. These blooms pose potential health risks for recreation, and impact the treatment of drinking water. Understanding the timing and distribution of the blooms may aid in planning by local communities and resources managers. Satellite data provides a means of examining spatial patterns of the blooms. Data sets from MERIS (2002–2012) and MODIS (2012–2014) were analyzed to evaluate bloom patterns and frequencies. The blooms were identified using previously published algorithms to detect cyanobacteria (~25,000 cells mL^−1^), as well as a variation of these algorithms to account for the saturation of the MODIS ocean color bands. Images were binned into 10-day composites to reduce cloud and mixing artifacts. The 13 years of composites were used to determine frequency of presence of both detectable cyanobacteria and high risk (>100,000 cells mL^−1^) blooms. The bloom season according to the satellite observations falls within June 1 and October 31. Maps show the pattern of development and areas most commonly impacted during all years (with minor and severe blooms). Frequencies during years with just severe blooms (minor bloom years were not included in the analysis) were examined in the same fashion. With the annual forecasts of bloom severity, these frequency maps can provide public water suppliers and health departments with guidance on the timing of potential risk.

## 1. Introduction

**Figure 1:**
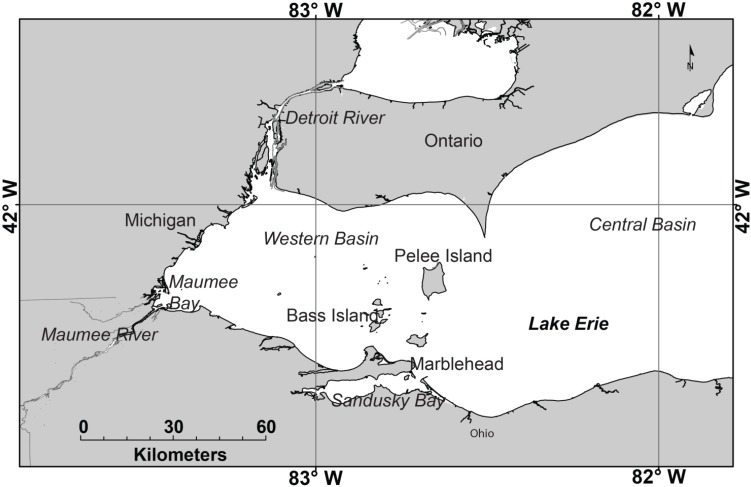
Study area and geographic features described in the text.

**Figure 2:**
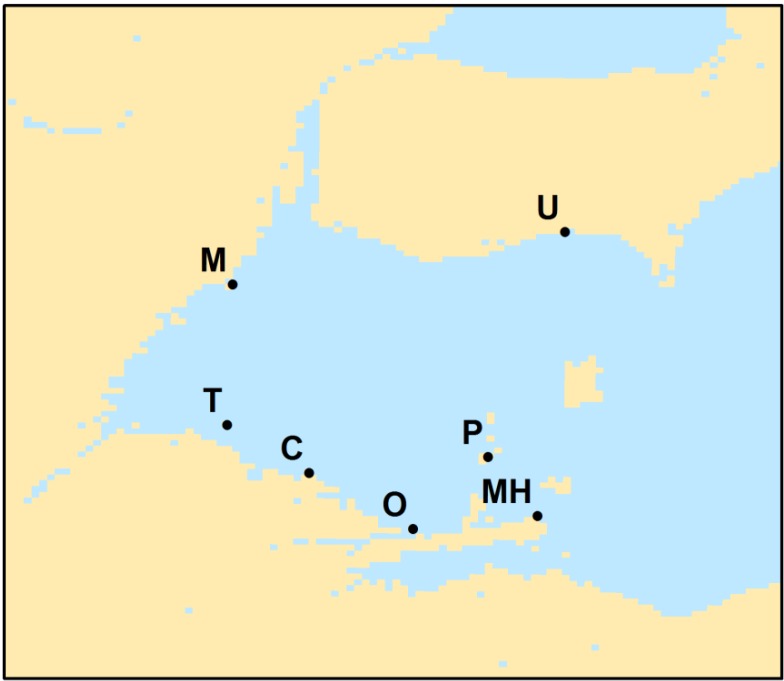
Location of Lake Erie municipal water intakes lettered as following: T = Toledo PWS; M = Monroe; C = Carroll Water and Sewer; O = Ottawa County Regional; P = Put-In-Bay Village PWS; MH = Marblehead Village PWS; U = Union.

Lake Erie (Figure 1) has experienced a recurrence of blooms with potentially toxic cyanobacteria this century [1], with six of the last seven years having significant blooms [2]. The dominant species of cyanobacteria in Lake Erie is *Microcystis aeruginosa* (henceforth referred to as *Microcystis*). *Microcystis* typically forms dense monospecific (single species) blooms, although *Anabaena*, *Planktothrix*, and other genuses of cyanobacteria may sometimes appear. These blooms have a variety of detrimental impacts, such as: taste and odor issues in municipal water supplies, potential human health issues, mortalities in domestic and wild animal populations, and adverse economic impacts in local communities [3,4]. Contamination of drinking water is a potential hazard, given the number of intakes around the western lake. In September, 2013, Carroll Township, Ohio (Station C on Figure 2), which supplies water to several thousand people, shut down its municipal water supplies for two days [5] owing to microcystin—the toxin found in *Microcystis*—concentrations greater than the World Health Organization guideline of 1 μg L^−1^ [6]. In August, 2014 microcystin concentrations reached the same risk level in the processed water of the city of Toledo, resulting in a two-day “do not drink” statement from its municipal water suppliers (station T on Figure 2) to approximately half a million customers [7].

NOAA has routinely issued short-term (<1 week) forecasts in Lake Erie since 2009 [2,8]. The demand for these forecasts has been high. The subscriber list for the Lake Erie forecast has experienced an annual growth rate of approximately 250% from its inception in 2009. More recently, NCCOS has started issuing seasonal forecasts [9,10,11] of cyanobacteria based on models previously presented [1]. A determination of the frequency of blooms over the 13 years of satellite data will provide a better understanding of timing and distributions of these potentially toxic blooms.

These frequencies may allow for spatial-temporal forecasts, which may be beneficial in both a micro and macroeconomic scale. The results could support planning by managers of public water suppliers and parks. They may also ultimately aid the public in avoiding contact with potentially toxic (and unaesthetic) cyanobacteria.

## 2. Results

### 2.1. Frequency Maps

**Figure 3:**
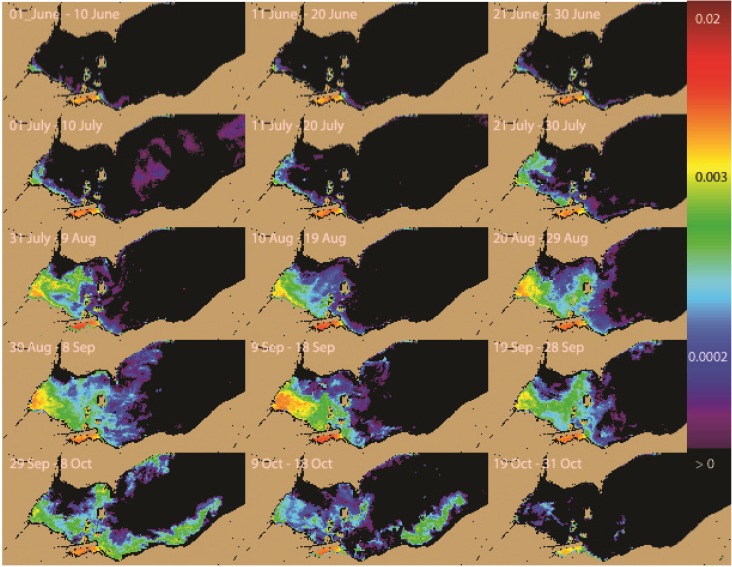
The average Cyanobacterial Index concentration of the 13 years (log scaled) for each 10-day period. Cell concentration can be estimated from the CI by Cells (mL^−1^) = 10^8^ × CI [1]. CI > 0.001 exceeds the WHO [6] threshold of 10^5^ cells mL^−1^.

The mean concentration of the cyanobacteria Index (CI) over the 13 years (Figure 3) shows the pattern of high concentration through the season. (The color scale is logarithmic, so the orange-red colors have 10-fold greater concentration than cyan colors.) Sandusky Bay has the highest consistent concentration, with little change through the season. This is typically a bloom from the cyanobacterium, *Planktothrix* [12]. The Maumee Bay and southwestern area of the western Lake Erie basin (WLEB) have the next highest mean concentration, with rapid increase between July 21 and August 09. In contrast, the northwest area, in the plume of the Detroit River, does not have a detectable concentration. Away from the Detroit River, low concentration may be present along the Ontario coast in early season, increasing somewhat into September.

The central basin shows two events; presence of cyanobacteria in July (July 01–10) and in early October. The July mean was produced by blooms that occurred in 2012 and 2013. The October mean owes to the severe bloom of 2011 (see the frequency discussion below).

**Figure 4:**
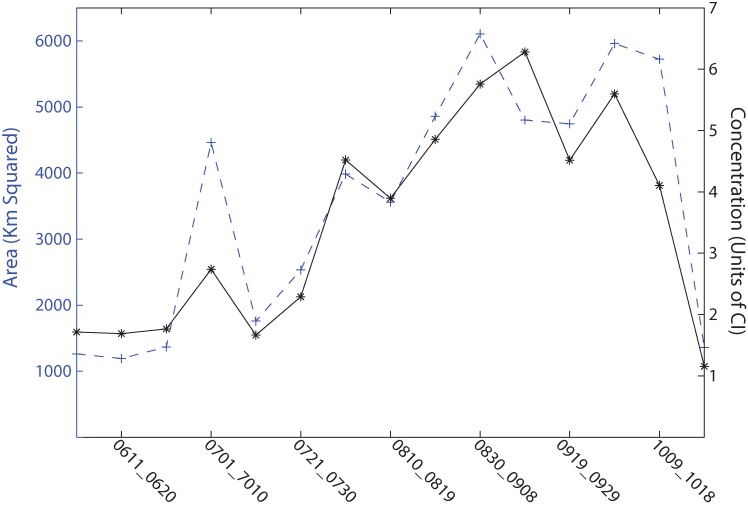
The 13-year average of the area and biomass in Lake Erie flagged by the satellite imagery for each 10-day period (0611 is June 11, *etc.*). Area is shown in blue, accumulated biomass is shown in black. The biomass is the accumulated biomass across the entire lake following previously published methods [1]. 1 CI is nominally 10^20^ cells. This is integrated spatially, thereby corresponding to biomass.

Accumulating the biomass across lake, including Sandusky Bay (Figure 4), provides a measure of the timing of the bloom development. The minimum value on June 1 reflects the presence of a bloom in Sandusky Bay, which persists through the season. The variability above this value captures the average bloom growth in the lake proper. Early July shows the short-lived bloom in the central basin. In the WLEB, development starts by July 22 on average, and peaks in area and biomass between August 30 and September 18. Overall, the peak lasts for 40 days (*i.e.*, four 10-day periods) before decreasing rapidly in October.

**Figure 5:**
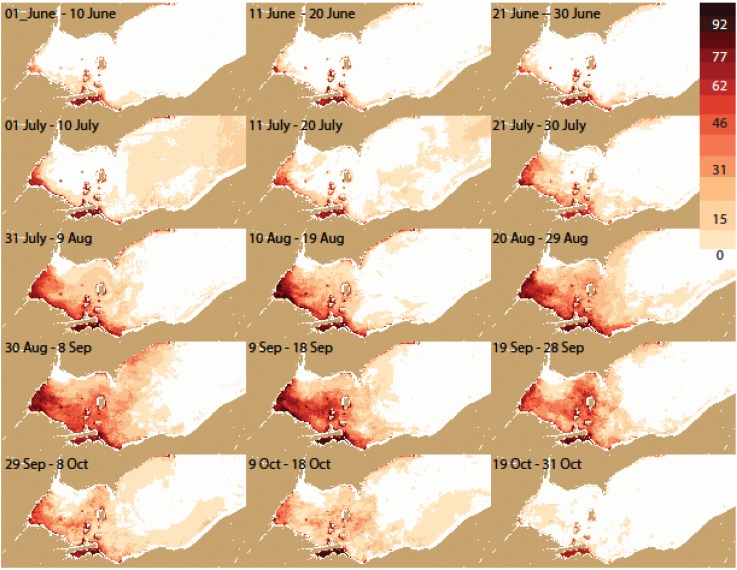
The spatial pattern (by pixel) of percentage frequency of detectable cyanobacteria. Analysis for each 10-day period during all years from 2002–2014.

**Figure 6:**
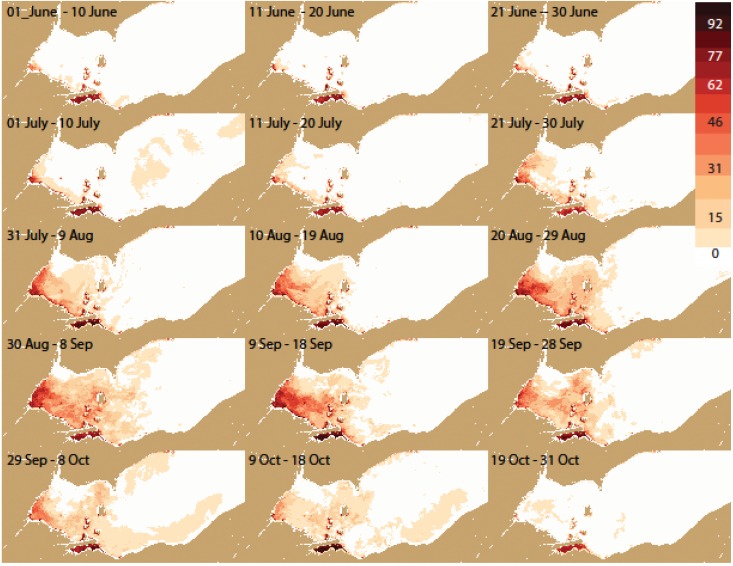
The spatial pattern of percentage frequency of severe cyanobacteria (>10^5^ cells mL^−1^, CI > 0.001) for each 10-day period during all years from 2002–2014.

**Figure 7:**
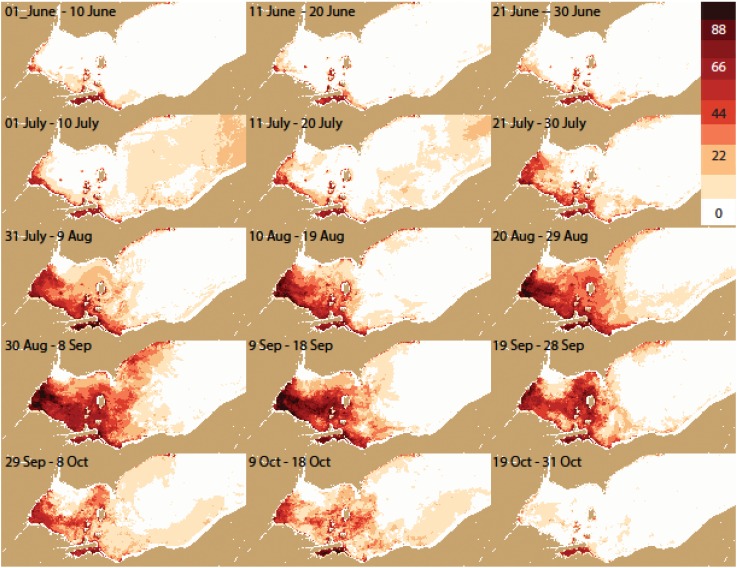
Same as Figure 5 for only years with blooms (percentage frequency of detectable cyanobacteria).

**Figure 8:**
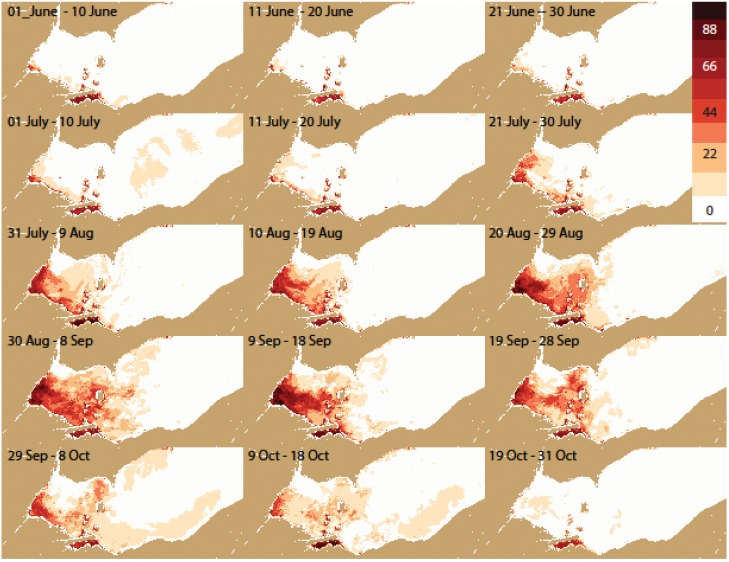
Same as Figure 6 for only years with blooms (percentage frequency of severe cyanobacteria).

The frequency distribution maps (Figure 5, Figure 6, Figure 7 and Figure 8) capture key aspects of bloom development. A persistent cyanobacteria bloom is present in Sandusky Bay which is typically of the genus *Planktothrix*. Generally, the western basin blooms start in Maumee Bay, with high frequencies in the southwest corner of the WLEB at the beginning of July. The frequency is greatest in the west, with high frequency expanding eastward over the season. The greatest extent of cyanobacteria presence is from August 30 to September 18. The cyanobacteria then become slightly less prevalent during the next 30 day period, before experiencing a relatively sharp decline in abundance during the 19 September–28 September period. Cyanobacteria are no longer present over the western basin of Lake Erie after 18 October.

In detail, the bloom is most common first along both the west (Michigan) and south (Ohio) shorelines. However, on the Michigan coast, blooms do not occur north of Monroe (Station M on Figure 2). This area is under the influence of the Detroit River, the large volume of water keeps the bloom back near the Maumee River (discharge of the Detroit River is ~35 times that of the Maumee River [13]).

Eastward movement is not uniform. Detectable concentrations occur relatively early along the Ohio coast to Marblehead (early July). Later in the season, the pattern changes and the greatest frequency of detectable or intense blooms is near the islands in September. Ottawa County (Figure 5 station O) has about half the frequency of blooms as do the islands. Generally, the chances of encountering cyanobacteria are less than 50% until August for the island region, while the peak frequency occurs between 9 September and 18 September for the western islands (*i.e.*, Bass), and between 19 September and 28 September for the. Pelee Island region.

### 2.2. Ontario Shoreline

The northern shoreline (Ontario) as a general rule is much less impacted relative to the southern shore (Ohio). The area east of Pelee Point is generally unaffected by cyanobacteria, with the only incident in the area occurring in 2011, the largest bloom in presented in the dataset show here [14]. The area between Pelee Point and the Detroit River Plume is more regularly affected and, like the Ohio shoreline, blooms are more likely to be encountered from 20 August though 28 September.

### 2.3. Drinking Water Supplies

**Figure 9:**
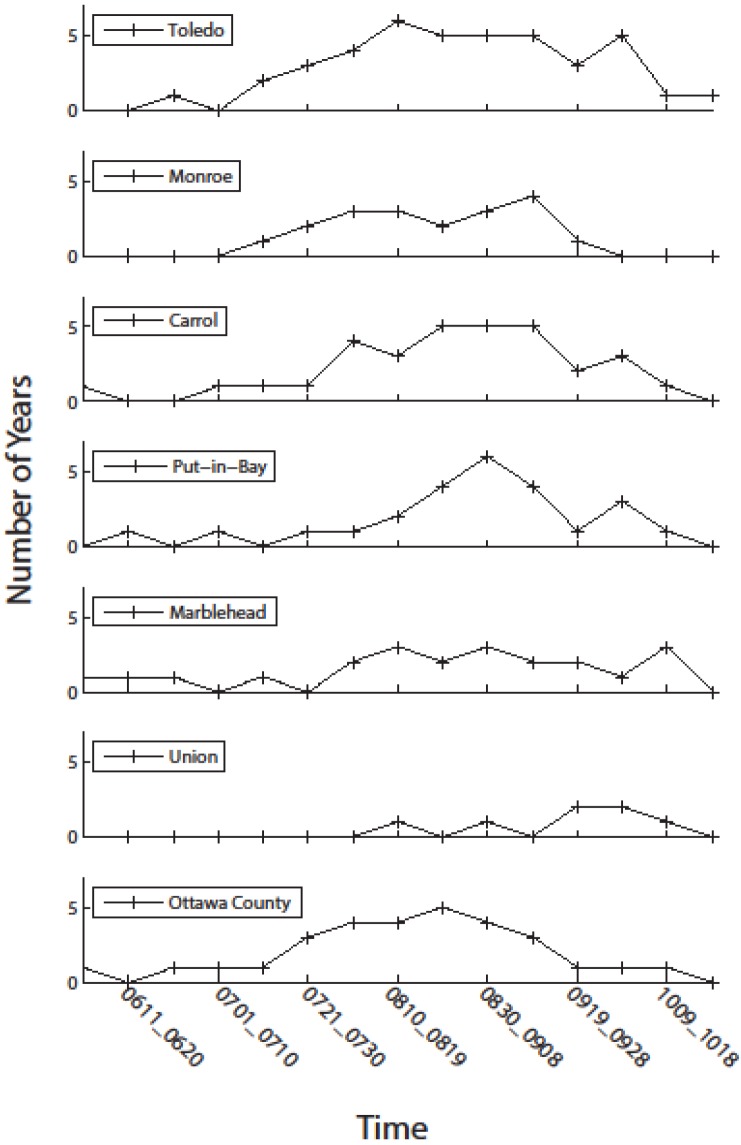
Frequency of severe blooms during the 2002–2014 record at the approximate location of selected water treatment intakes from Figure 2. Except for Toledo station (station 1), the data from the other stations were taken 2 pixels (~2 km) into the center of the lake to obtain valid data without land contamination or masking.

As most intakes are within one km of the shore, we examined the frequency patterns of CI > 0.001 at three km offshore near several intakes. These water intake facilities covered a relatively large disparate area of western Lake Erie, with one station in Ontario, one in Michigan, and the remaining five in Ohio. The timing of risk for water suppliers varies (Figure 9). Toledo has the highest frequency, with 70 days having 5–6 years of intense blooms. Monroe, in Michigan, is on the edge of the influence of the Detroit River plume, and has significantly less bloom activity most years relative to Toledo. Moving eastward, there is a difference with the mainland when compared to the islands. Carroll and Ottawa County have early blooms, while Put-in Bay (Bass Island) has less frequent blooms in early August, with a short peak of high frequency at the end of August. It should be noted that the CI can only be used as a proxy for cyanobacteria biomass and not the toxin concentration. The biomass and the toxin concentration may covary, but do not necessarily do so.

## 3. Discussion

Creating two frequency maps based on bloom years and all years is based on the value of the seasonal predictions issued by NOAA [9,10,11]. In these seasonal predictions, NOAA predicts whether a bloom of cyanobacteria is to be expected in Lake Erie based on a statistical model using discharge of total phosphorus concentration from the Maumee River as outlined previously [1]. If a bloom is to be expected, the frequency map using just the bloom years would be more likely to be an accurate assessment of the probability map of the bloom relative to the frequency map using all years.

Distinguishing between bloom and non-bloom years allows for application of the annual forecasts [9,10,11]. A detailed method to calculate summer peak *Microcystis* biomass using spring discharge from the Maumee River has been presented elsewhere and is appropriate to use for this application [1]. This has been used as the basis of a forecast issued annually by NOAA since 2012. The previous year’s forecast is validated prior to the new forecast being issued. Thus far the accuracy of the forecast has been well received by users [15], and the forecast will continue to be issued.

The maps can assist natural resource managers as they plan on mitigation for the blooms. The municipalities that use Lake Erie for drinking water can make plans to avoid intake issues during times when blooms are likely to be present, or to plan for supplies to treat water to mitigate the risk to drinking water. Sampling of parks and public beaches for toxins can be made more strategically, as well. Even the public can use the maps to plan recreational activities to gain maximum use of the lake, while reducing risk. This could have positive impacts to the local economy as it would encourage repeat visitors if negative experiences can be avoided. Furthermore, actual mitigation of blooms may become possible if it is known when and where they will occur.

The distribution has provided insight into the patterns of the blooms. The contrast between the area near the Maumee River and the Detroit River is striking. It has been shown that the phosphorus load from the Maumee River drives the blooms to a large extent [1]. The results here show that the plumes are located in most years in the area of the Maumee River. In contrast, the blooms do not occur near the Detroit River. The pattern in the center of the WLEB, which has relatively high frequency of blooms, likely results from the transport of the bloom around the Detroit River plume. The blooms do not make landfall on the northern Ontario coast until far east of the Detroit River. The large difference in nutrient concentration between the Detroit River and Maumee River explains this difference; the Detroit River has less than 1/20 of the mean concentration of phosphorus of the Maumee River [13]. The disparity of the hydrodynamics is not necessarily an issue as the Detroit River discharge is ~35 times higher than the Maumee River discharge [13,16].

The forecast could also be useful for educational purposes by informing the public when and where blooms may occur and what causes them. The blooms have a tendency to congregate in harbors and on beaches where they are more likely to be encountered by the public [16]. These areas would be easier for short term mitigation of the blooms relative to the large open areas of the Lake. Mitigating the inshore areas affected by blooms would partially alleviate the local economic impacts from the blooms. The public also perceives the Lake as being polluted when it encounters blooms of cyanobacteria, and by applying short term mitigation techniques it may be possible to raise the public perception of the lake ecosystem.

Ecologically speaking, these frequency maps serve another purpose. Cyanobacteria blooms are common in western Lake Erie. The analysis in Figure 4 shows that the maximum intensity (biomass) of the blooms occurs between 9 September and 18 September. These maps give a spatiotemporal timeframe on the initiation and senescence of the blooms, which was not previously available. Furthermore, it gives a likelihood of where the bloom will likely next spread once it is underway. For instance, it seems highly probable that the blooms start in Maumee Bay, and will spread from there. In nearly all years, the bloom was essentially gone by 31 October, with the exception of Sandusky Bay.

## 4. Methods

The delineation and detection of these blooms has been well-documented with satellite ocean color data [1,2,8,17,18,19,20]. The Medium Resolution Imaging Spectrometer (MERIS) on board the Envisat-1 satellite provided data for the summers from 2002–2011. On April 8, 2012 Envisat failed, resulting in a cessation of MERIS data. The Moderate Resolution Imaging Spectroradiometer (MODIS) was used for 2012–2014. MERIS level 2 reflectance (R; with sr^−1^ units) data sets from the second reprocessing were obtained from the European Space Agency (ESA). MODIS was obtained as level 0 data from the National Aeronautics and Space Administration (NASA) and processed to Rayleigh corrected bi-directional reflectance (ρ_s_; which is dimensionless) using NASA’s SeaDAS package, with the “rhos_s” option in SeaDAS l2gen. Products were processed in equal area Sinusoidal projection, with 1.1 km pixel scale through 2013, and were processed to Albers 1.1 km equal area projection in 2014. All sinusoidal images were reprojected to the Albers equal area projection for all analysis (as all future analyses in our group will use the Albers projection).

Both data sets were then processed with equivalent spectral shape (second derivative) algorithms, based around 680 nm [17,20]. With MERIS bands the equation is:

(1) $$S_{2\text{d}}(681)=R(681)-R(665)-\{R(709)-R(665)\}\frac{\left(681-665\right)}{\left(709-665\right)}$$

where R is the reflectance and the values are the band centers. For MODIS, the MERIS algorithm was adjusted to the MODIS Aqua sensor [20] to yield:

(2) $$S_{2\text{d}}(678)={\text{ρ}}_{s}(678)-{\text{ρ}}_{s}(667)-\{{\text{ρ}}_{s}(748)-{\text{ρ}}_{s}(667)\}\frac{\left(678-667\right)}{\left(748-667\right)}$$

where ρ_s_ is Rayleigh corrected bi-directional reflectance.

For MERIS, the cyanobacterial index (CI) is found from [17]:

*CI* = −*S*_2d_(681) (3)

and for MODIS the conversion to match MERIS [20] is:

(4) $$C\text{I}=-S_{2\text{d}}(678)\times 1.3$$

where the CI has units of sr^−1^.

The time series shown here extends from 2002–2014. The portion of the time series covering 2002–2011 used MERIS imagery, and the portion covering 2012–2014 used MODIS imagery. MERIS produced superior results with less noise than MODIS and no saturation. MODIS saturates for bright pixels, which can result from glint, haze, and turbid water [21,22,23]. These conditions can occur during severe algal blooms during the summer [20], resulting in failure of the CI calculation expressed in Equation (4). While the time-series from 2012 showed a bloom, the imagery was not overly turbid and the MODIS bands used in Equation (2) did not saturate in bloom areas, so the MODIS saturation was not an issue. However, the 2013 and 2014 blooms were more intense and highly reflective, resulting in some saturation. A mechanism was needed to quantify the biomass under saturation. The “land” bands in MODIS are calibrated in such a way that they will not saturate even under the most turbid water conditions, and have been recommended for use when the nine bands commonly used for ocean color from MODIS (covering 412–869 nm) have saturated [21]. Cyanobacterial blooms are detectable as bright water, which can provide an estimate of presence and quantity of biomass [24,25]. The near-infrared (NIR) bands on the MODIS Aqua sensor were used to calculate a reflectance proxy for the CI for MODIS.

*CI*_sat_ = 0.5 * [ρ_w_(859)]^0.5^ (5)

with

ρ_w_(859) = ρ_s_(859) − ρ_s_(1240) (6)

where the 1240 band is used as a nominal atmospheric correction [23]. The derivation of Equation (5) was empirically tuned with a simple root relationship to overlap the retrieved CI values around saturated pixels. Slight errors in the tuning of Equation (5) are not important to the study, as they would apply when conditions are well above the “severe bloom” threshold discussed below. While scum or algae floating on the surface can produce saturation in the MODIS bands, saturation also occurs in areas without surface scums. As a result, using other metrics like the “floating algae index” [26] would still require yet another algorithm (like Equation (5)) to provide coverage of all saturated areas.

When saturation did not occur, the standard CI solution (Equation (4)) was used; for the conditions when saturation occurred within a bloom, the CI_sat_ from Equation (5) was applied. A tuning of reflectivity to biomass would probably vary between years, depending on the bloom characteristics, like cellular chlorophyll content. Resuspended sediment is uncommon during the summer in Lake Erie, and only the saturated pixels contained within blooms (areas of CI) were used. In 2014, the scattering appeared slightly milder, so the correction of Equation (6) was reduced proportionately.

CI varies linearly with biomass, with a value of 10^−3^ sr^−1^ corresponding to 10^5^ cells mL^−1^ [1], which is the World Health Organization’s (WHO) threshold of significantly increased risk for human health [6]. The minimum detection of the CI is still being assessed; however, a CI of 2 × 10^−4^ sr^−1^ produces consistent retrievals of the bloom edge over multiple images for both sensors, indicating that the minimum detection is less than 20,000 cells mL^−1^, which is also the recommended threshold for avoiding irritative effects [6].

Clouds were masked and 10-day composites were made for each year during the bloom period using the maximum value of the CI at each pixel. There are several advantages to utilizing maximum value composites. The first advantage is that the composite reduces cloud interference, reducing the data to a systematic set of generally cloud-free images. The second key advantage is to estimate areal biomass. When winds are strong (>7.7 m s^−1^, or stress of 0.1 Pa), the bloom is mixed through the water column, diluting the surface concentration [18,27]. Under calm winds, however, *Microcystis* floats upward with dense accumulations visible on the lake [28]. The surface concentration (CI) estimated from satellite during calm conditions then represents the *Microcystis* that is present in the water column [18], however, the concentration detected during high winds underestimates true biomass. Typically, during any 10-day period in the summer, there is a period of calm clear weather [8], allowing this estimate. The cells return to the surface within 24–48 hours after a wind event. The bands used for the algorithm quantify concentration within one meter of the surface in the clearest water [29], less as turbidity increases (usually because of the bloom). Finally, using a 10-day composite makes biological sense as the doubling time for *Microcystis* is as low as 10 days in the Great Lake region [30].

Blooms in Lake Erie generally occur in the summer when water temperatures exceed 15 °C, although blooms can persist in cooler waters once established [8]. As a result, the bloom season considered here is defined as 1 June through 31 October following conventions published elsewhere [1]. Fifteen separate 10-day composites covering the bloom year (1 June to 31 October) were constructed from methods detailed in elsewhere [1]. The final 10-day composite actually consists of 13 days to complete October (to October 31; See Table 1).

**Table 1:** Shown here are the start and end dates of each of the 15 10-day composites discussed in the text.

Composite number	Start date	End date
1	01 June	10 June
2	11 June	20 June
3	21 June	30 June
4	1 July	10 July
5	11 July	20 July
6	21 July	30 July
7	31 July	9 August
8	10 August	19 August
9	20 August	29 August
10	30 August	8 September
11	9 September	18 September
12	19 September	28 September
13	29 September	8 October
14	9 October	19 October
15	19 October	31 October

With the 10-day composites in hand, several climatological data sets were generated. The means for each of the 15 separate 10-day composites were made from the averages of all the years.

Frequency maps were made across two sets of conditions: (1) all years or bloom years, and (2) all detectable (measurable CI) and CI > 0.001. As noted previously, CI = 0.001 corresponds to the WHO significantly increased risk threshold of 10^5^ cells mL^−1^. The lower threshold indicates presence of cyanobacteria at a level that poses some (but slight) risk. Bloom years are those that had significant blooms. Negligible blooms were detected in 2002 and 2005–2007 [1,31]. While 2012 had a small bloom [2], it was locally dense and nearly equivalent to the 2004 bloom, and is included. As a result, the frequency maps were calculated just for years containing defined blooms (2003, 2004, 2008–2014). These frequency maps were made based on all bloom types during just the bloom years.

Spurious pixels due to satellite mis-navigation, cloud edges, and mixed land-water pixels were removed from analysis. It should be noted that the two pixels adjacent to the coastline in the southern shore of Lake Erie had to be masked due to somewhat severe land interference issues. These were caused mostly by mis-navigation, although in the case of MODIS, slow sensor response as the sensor scanned from land, where it always saturated, onto water was the cause. In individual MERIS scenes that do not have these issues, the nearshore, masked, pixels appear to have similar concentrations to the offshore pixels. Still, the concentration can vary nearshore, particularly with light winds moving surface scums. The frequency data sets are available in the journal’s supplementary data section.

## 5. Conclusions

The methods used here give an approximation of the spatiotemporal cyanobacterial quantification for western Lake Erie. The frequency maps can be updated as more years of data are available from MODIS. In 2015, the European Space Administration is planning on launching the replacement for MERIS, the Ocean Colour Land Imager (OLCI) sensor, on board the Sentinel-3 satellite. The accumulation of data will lead to increased statistical power of the frequency maps and allow for evaluation of them as tools for predicting bloom position and timing. The frequency information can allow managers to anticipate the timing of the arrival and duration of the bloom in their area when the seasonal forecast is made. This information allows planning for sampling, supplies and resources, and strategic monitoring to protect public health.

## Supplementary Materials

Supplementary materials can be accessed at: http://www.mdpi.com/2072-6651/7/5/1649/s1.
