# Supplementary material for: Spatial and Temporal Patterns in the Seasonal Distribution of Toxic Cyanobacteria in Western Lake Erie from 2002–2014
Source: Toxins (Basel). 2015 May 12;7(5):1649–63. doi: 10.3390/toxins7051649 (PMC4448166; doi:10.3390/toxins7051649)
Supplement: Supplementary file 1 [file toxins-07-01649-s001.zip › readme.docx]

THIS DATA SHOULD ONLY BE USED FOR LAKE ERIE, NOT LAKE ST. CLAIRE!

The comp* files are the 10 day composites.

Comp = composite

The next 4 are the start month and day followed by the end month and day.

The scaled numbers can be brought to CI units by:

CI = 10^(SCALED_NUMBERS/100-4)

The frequency files show the number of years that exhibited a CI > 0 or greater the 0.001 depending on the file name.

There are 4 different types of frequency files.

(**1**) BLOOMyrs_frequency*greater_0.001.tif are the files corresponding to severe blooms. The numbers are the number of years that the CI was greater than 0.001. 253 is land. Boom years are as follows: 2003, 2004, 2008–2014

(**2**) (1) BLOOMyrs_frequency.tif are the files corresponding to any bloom. The numbers are the number of years that the CI was greater than 0. 253 is land. Boom years are as follows: 2003, 2004, 2008–2014

(**3**) frequency*greater_0.001.tif are the files corresponding to severe blooms. The numbers are the number of years that the CI was greater than 0.001. 253 is land. Data uses years from 2002–2014.

(**4**) frequency*.tif are the files corresponding to any bloom. The numbers are the number of years that the CI was greater than 0. 253 is land. Data uses years from 2002–2014.

The images use an Albers equal area projection.

Spurious features were removed from analysis. See Wynne and Stumpf, 2015.
